# Alkaline extract of the seaweed *Ascophyllum nodosum* stimulates arbuscular mycorrhizal fungi and their endomycorrhization of plant roots

**DOI:** 10.1038/s41598-021-93035-9

**Published:** 2021-06-29

**Authors:** Sarah Hines, Timo van der Zwan, Kevin Shiell, Katy Shotton, Balakrishnan Prithiviraj

**Affiliations:** 1 Marine Bioproducts Research Laboratory, Department of Plant, Food and Environmental Sciences, Dalhousie University, Truro, NS Canada; 2 Acadian Plant Health, Acadian Seaplants Ltd., Dartmouth, NS Canada

**Keywords:** Plant development, Plant sciences, Plant symbiosis, Arbuscular mycorrhiza

## Abstract

*Ascophyllum nodosum* extracts (ANE) are well-established plant biostimulants that improve stress tolerance and crop vigour, while also having been shown to stimulate soil microbes. The intersection of these two stimulatory activities, and how they combine to enhance plant health, however, remains poorly understood. In the present study, we aimed to evaluate: (1) the direct effect of ANE on the arbuscular mycorrhizal fungus *Rhizophagus irregularis*, and (2) whether ANE influences endomycorrhization in plants. ANE enhanced development of *R. irregularis *in vitro, showing greater spore germination, germ tube length, and hyphal branching. Greenhouse-grown *Medicago truncatula* drench-treated with ANE formed mycorrhizal associations faster (3.1-fold higher mycorrhization at week 4) and grew larger (29% greater leaf area by week 8) than control plants. Foliar applications of ANE also increased root colonization and arbuscular maturity, but did not appear to enhance plant growth. Nonetheless, following either foliar or drench application, *M. truncatula* genes associated with establishment of mycorrhizae were expressed at significantly higher levels compared to controls. These results suggest that ANE enhances mycorrhization through both direct stimulation of arbuscular mycorrhizal fungus growth and through stimulation of the plant’s accommodation of the symbiont, together promoting the establishment of this agriculturally vital plant–microbe symbiosis.

## Introduction

Seaweed extracts, such as those of the brown seaweed *Ascophyllum nodosum*, have a long history of use as plant biostimulants^1^. *A. nodosum* extracts (ANE) have been demonstrated to enhance plant tolerance to biotic and abiotic stresses through both supplementation of stress-protective compounds taken up by the plant and through elicitation of systemic responses^2–4^. It is also becoming clear that seaweed extracts can indirectly influence plant health through beneficially impacting the structure and function of the plant microbiome, such as through enrichment of microbial taxa comprising mutualistic symbionts and other microbes that promote plant growth and soil nutrient cycling^5,6^.

The rhizosphere is occupied by millions of microbes that are intimately connected to the health of a plant^7^. The microbiota surrounding plant roots are actively cultivated through exudation of up to 20–40% of a plant’s photosynthate into the rhizosphere to support microbially assisted nutrient acquisition^8^. One of the most pervasive groups of microbes found in the rhizosphere, as well as the endosphere, are the arbuscular mycorrhizal fungi (AMF), obligate endosymbionts associated with more than 80% of land plants^9^. AMF facilitate the uptake of nutrients by effectively extending the reach and increasing the surface area of a plant’s root system through an interconnected extraradical mycelium that interfaces with the plant at the periarbuscular membrane for reciprocal nutrient exchange, while also influencing the plant’s secondary metabolism^10^ and offering protection from a wide range of abiotic and biotic stresses^11^. The metabolism of soil-borne AMF can be stimulated by plant root secondary metabolites such as strigolactones and coumarins, serving as molecular signals that induce spore germination and hyphal growth to recruit AMF for eventual intracellular symbiotic accommodation^12,13^. Agricultural crops in association with AMF may display enhanced growth and yield stability^14,15^, and perhaps more critically, contribute to increased agricultural sustainability through contributions to soil structure via AMF-mediated soil aggregation, and through enhanced nutrient acquisition in the soil that can facilitate reduced fertilizer usage, providing ecosystem and financial benefits even in the absence of yield increases^16^.

A growing body of evidence demonstrates the beneficial influence of ANE on plant-associated microbiota, along with correlated benefits to plant health and yield^5,17,18^. In this prior work, applications of ANE have been shown to broadly influence the structure and activity of microbial communities in the rhizosphere, increasing rhizosphere biodiversity, respiration, and metabolic activity. However, research into the influence of seaweed extracts on specific microbes, such as AMF, and the influence on their interactions with plants, remains limited. Kuwada et al. demonstrated a direct stimulatory effect of methanolic extracts from red and green seaweeds on AMF hyphal growth, as well as stimulation of plant colonization when applied to the soil^19^. Earlier work showed similar beneficial results on AMF growth and colonization following soil application of methanolic extracts of the brown seaweeds *Laminaria japonica* and *Undaria pinnatifida*^20^. A recent study showed that the combined drench application of neutral aqueous extract of *Padina gymnospora* in combination with *Rhizophagus irregularis* spores resulted in enhanced colonization and growth of tomato plants^21^. While methanolic extracts and neutral aqueous extracts of these seaweed species have been demonstrated to stimulate AMF growth and colonization, it remains unknown whether alkaline extracts of *Ascophyllum nodosum* possess similar bioactivity. This is despite the fact that commercial production of seaweed-based plant biostimulants predominantly employs aqueous alkaline extraction, and despite *A. nodosum* being one of the most abundantly used seaweed species industrially^22^. Seaweed extracts are also regularly applied foliarly in the field, yet thus far there have been no studies reporting the influence of foliar applications of seaweed-based biostimulants on AMF colonization. Through elicitation of a systemic response, foliar applications could potentially be expected to modulate root exudation and affect the establishment of plant–microbe associations^23,24^.

In this study, we examined the effect of aqueous alkaline ANE on spore germination and development of the agriculturally important AMF species *Rhizophagus irregularis*. Further, in a greenhouse trial using the model legume *Medicago truncatula*, we studied the effect of foliar and drench applications of ANE on the colonization of *R. irregularis*, interrogated through root microscopy and expression analysis of *M. truncatula* genes involved in the signaling for and accommodation of endosymbiotic fungi.

## Results

### ANE stimulates *R. irregularis* spore germination in vitro

**Figure 1:**
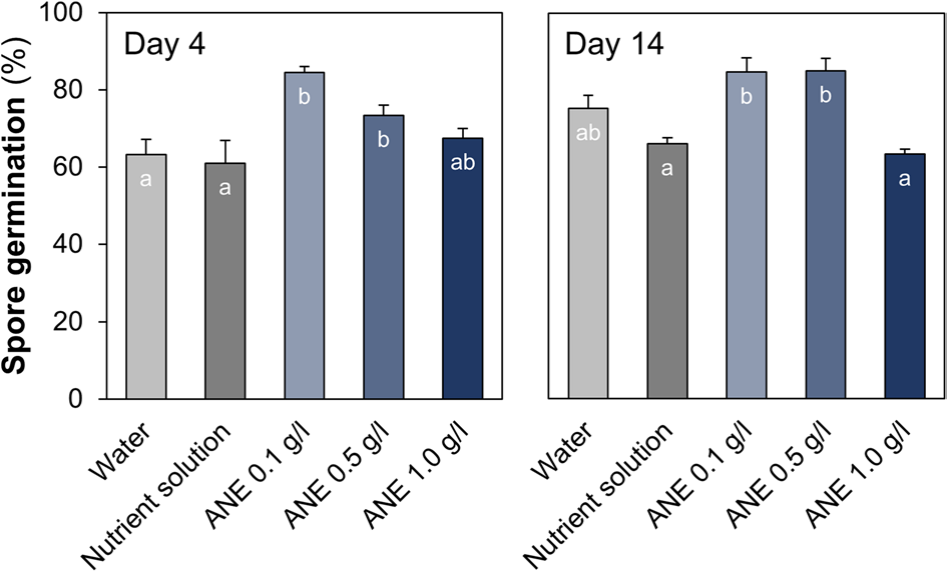
*R. irregularis* spore germination at days 4 and 14 grown on agar with treatments as labelled, each treatment comprising 6 replicate Petri dishes containing > 80 AMF spores (letters indicate Tukey’s post-hoc test significance groups, α = 0.05, following analysis of variance).

*R. irregularis* spore germination significantly improved in the presence of 0.1 and 0.5 g/l ANE at day 4 relative to the water and nutrient controls (Fig. 1). By day 14, significant differences remained compared to the nutrient solution control, but not the water control. Treatment with 1.0 g/l ANE on the other hand did not result in significant differences from the controls.

**Figure 2:**
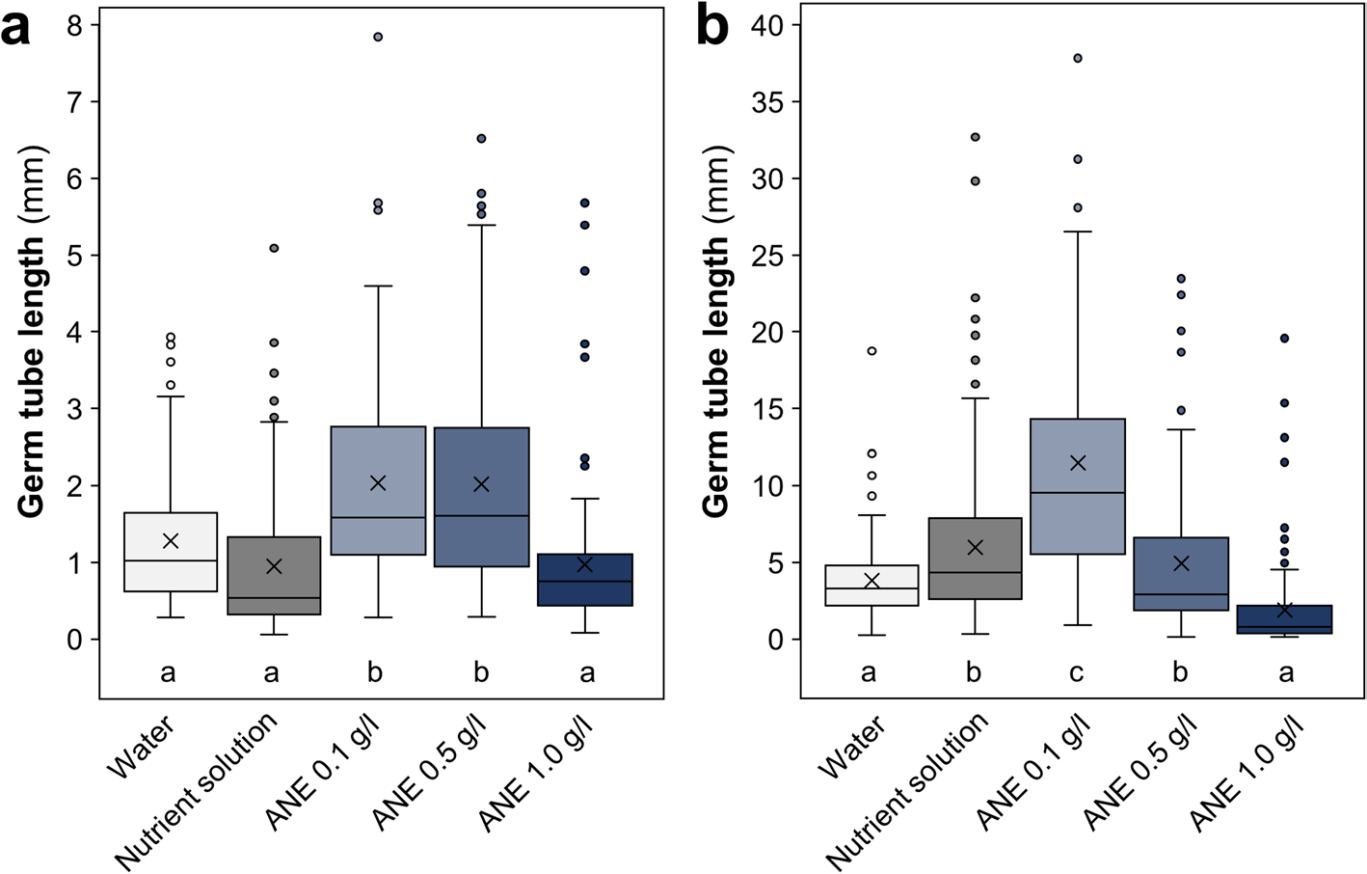
Germ tube lengths of germinated *R. irregularis* spores on agar with treatments as labelled after (**a**) 4 days and (**b**) 14 days. Letters indicate Tukey’s post-hoc test significance groups, α = 0.05, following analysis of variance; n = 100 per treatment; means are marked by × and medians by the horizontal line.

ANE treatment at rates of 0.1 and 0.5 g/l significantly increased *R. irregularis* germ tube length at day 4, compared to 1.0 g/l ANE and the controls, with an average 1.5-fold higher mean germ tube length (Fig. 2a). By day 14, only the 0.1 g/l ANE rate showed significantly higher germ tube lengths compared to both controls, attaining 1.9-fold and 3.0-fold higher mean germ tube length than the nutrient control and water control, respectively (Fig. 2b). Overall, the stimulation of AMF germ tube growth by ANE demonstrated a dose-dependent response, with a maximum at 0.1 g/l, of the ANE concentrations examined here.

**Figure 3:**
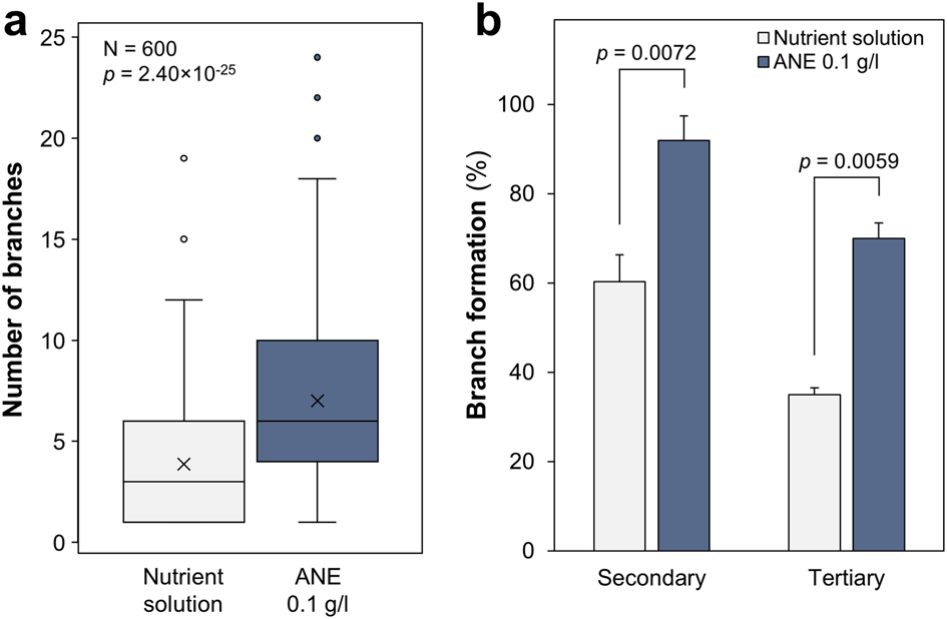
Branching of *R. irregularis* spore hyphae grown on agar with nutrient solution or 0.1 g/l ANE. Number of branches per spore (**a**) and the secondary and tertiary branch formation as a percentage of total spores (**b**) are shown. Per treatment, 300 germinated spores were assessed 4 days after spores were placed in the petri dishes. Means are marked by × and medians by the horizontal line in (**a**); indicated *p* values are from *t* tests, α = 0.05.

Given the considerable stimulation of spore germination with 0.1 g/l ANE, the influence of this ANE concentration on *R. irregularis* hyphal branching was examined in comparison to a nutrient control (Fig. 3). The ANE-treated spores showed significantly higher branching per spore compared to the control (Fig. 3a). Likewise, the percentage of spores forming secondary and tertiary branches was substantially higher following ANE treatment (Fig. 3b).

### ANE treatment improves *R. irregularis* colonization in *M. truncatula*

**Figure 4:**
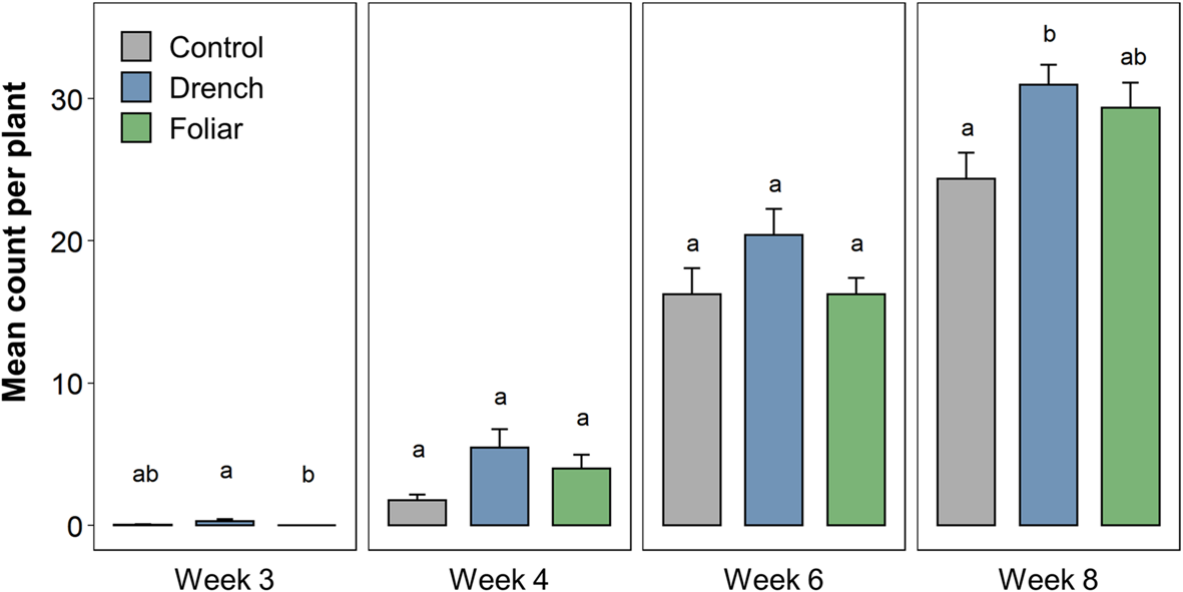
The number of root segments per *M. truncatula* plant maximally colonized by *R. irregularis* following treatment with ANE foliarly, drench or neither (control) through weeks 3 to 8 of greenhouse growth. Maximum colonization was defined as an intensity score of 5 and an arbuscule score of 3, with 50 root segments analyzed for 10 randomly selected plants per treatment per week. Letters indicate the significance groups determined via a Kruskal–Wallis test followed by a Dunn’s post-hoc test, *α* = 0.05.

**Figure 5:**
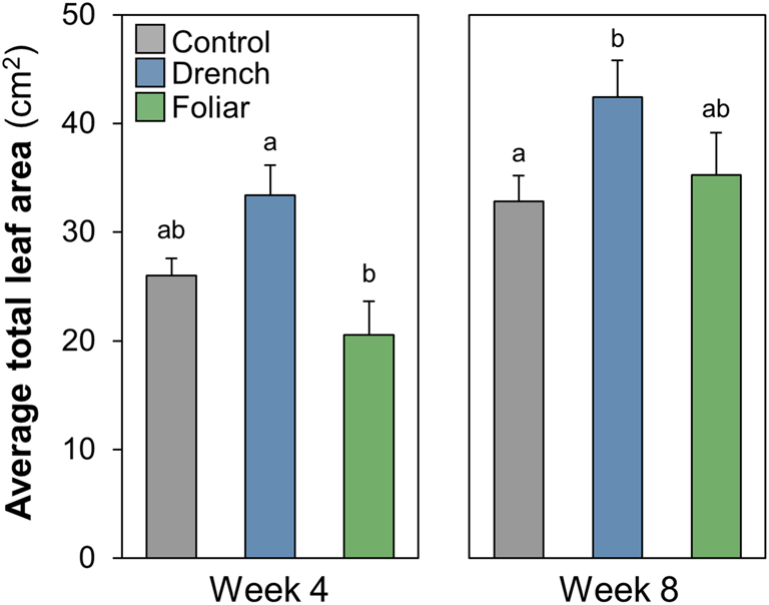
Average total leaf area of *M. truncatula* plants grown with *R. irregularis* inoculant, treated with nutrients only (control) or with ANE foliarly or via drench. Letters indicate Tukey’s post-hoc test significance groups, α = 0.05, following analysis of variance, with 10 plants measured per treatment.

To examine the influence of ANE treatment on the colonization of *R. irregularis* in *M. truncatula* following inoculation, roots were stained and examined microscopically, rating the progression of colonization based on colonization intensity (0–5) and arbuscular abundance (0–3)^25^ over the course of 8 weeks of greenhouse growth. Mature mycorrhization was assessed as a root segment having a colonization intensity score of 5 (> 90% colonization) combined with an arbuscule score of 3 (saturation). Across the weeks, drench-treated plants showed increased mature mycorrhization compared to the control (Fig. 4), albeit to a statistically significant degree only at week 8. At the earlier stages of colonization, drench-treated plants displayed 3.1-fold higher mean fully colonized root segments per plant at week 4 (*p* = 0.16), and remaining 1.3-fold greater at week 8 (*p* = 0.04). While not to a statistically significant degree (at an *α* of 0.05), foliarly treated plants showed 2.3-fold increased mature mycorrhization at week 4 compared to the control plants (*p* = 0.17), and remaining 1.2-fold greater at week 8 (*p* = 0.11). The enhanced colonization stimulated by ANE drench treatment was accompanied by enhanced plant growth, showing increased leaf area (Fig. 5). Leaf area of the foliarly treated plants did not differ to a statistically significant degree from the controls in either week.

### ANE treatment induces a transient modulation in AMF accommodation-related gene expression at the early stages of colonization

Given the positive influence of ANE on *R. irregularis* colonization and *M. truncatula* growth following drench or foliar treatment, we next sought to examine how the expression of *M. truncatula* genes related to fungal endosymbiont accommodation were affected by ANE treatments over the course of colonization. The expression of nineteen genes of interest (Table 1) representing genes previously demonstrated to be involved with precontact signaling, early establishment of the symbiosis, and/or arbuscule formation was examined^26,27^. Expression of the genes *DMI1*, *SBT1.2*, *ChitIII-2* remained near or below the limit of detection and these genes were not analyzed further.

**Table 1:** List of *M. truncatula* genes analyzed in this study and their functions in plant–arbuscular mycorrhizal fungus symbiosis.

Gene	Function	References
*ENOD11*	Putative cell wall repetitive (hydroxy)-proline-rich protein often used as a marker of early symbiosis establishment	^51^
*CHS1*	Chalcone synthase involved in flavonoid synthesis	^43^
*DMI2*	Plasma membrane receptor-like kinase involved in symbiont perception	^45^
*DMI1*	Nuclear ion channel required for early symbiosis signal transduction	^46^
*D27*	Beta-carotene isomerase involved in strigolactone synthesis	^44^
*IPD3*	Transcription factor that in concert with DMI3 activates a transcriptional response to AMF	^48^
*DMI3*	Calcium and calcium/calmodulin-dependent serine/threonine-protein kinase	^47^
*RAM1*	GRAS-domain transcription factor involved in mycorrhizal-specific signaling	^52^
*RAM2*	Glycerol-3-phosphate acyl transferase involved in lipid biosynthesis that enhances hyphopodia formation	^55^
*ExpB1*	Beta-expansin involved in prepenetration apparatus development	^49^
*ChitIII-3*	Acidic endochitinase putatively involved in the suppression of pathogen defence responses during AMF symbiosis	^60^
*ChitIII-2*	Acidic endochitinase putatively involved in the suppression of pathogen defence responses during AMF symbiosis	^59^
*STR*	Periarbuscular membrane-localized lipid transporter required for arbuscule development	^56^
*STR2*	Periarbuscular membrane-localized lipid transporter required for arbuscule development	^56^
*FatM*	AMF-specific palmitoyl-acyl thioesterase required for arbuscule development	^54^
*RAD1*	GRAS transcription factor required for arbuscule development	^53^
*PT4*	Phosphate transporter localized in the periarbuscular membrane, involved in acquisition of phosphate released by AMF	^57^
*VAPYRIN*	Ankyrin-repeat protein required for nutrient exchange at the periarbuscular membrane	^58^
*SBT1.2*	Subtilisin-like protease; high similarity to the *Lotus japonicus SBTM1* subtilisin that supports arbuscular development	^50^

**Figure 6:**
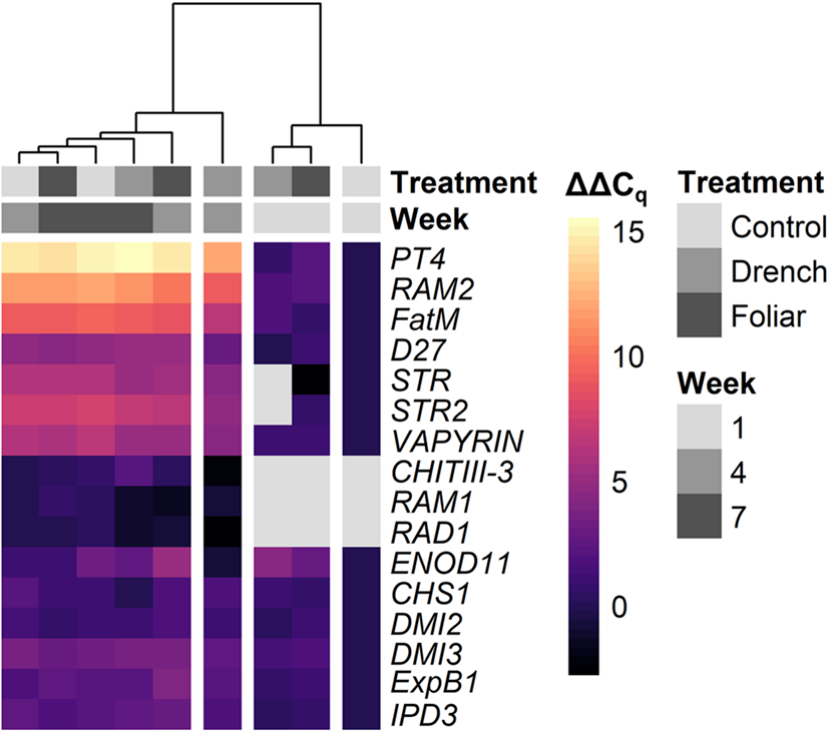
Heatmap of gene expression (ΔΔC_q_ values) in roots of *M. truncatula* plants treated with ANE foliarly or via drench in comparison to control treatments at weeks 1, 4 and 7 of growth following *R. irregularis* inoculation. Gray data points indicate expression levels below the limit of detection. The dendrogram shows hierarchical clustering of the treatments at each week calculated using the complete-linkage method. Expression was normalized to the control at week 1, or to the control at week 4 for genes where expression was below the limit of detection at week 1.

Clustering of expression profiles of the genes of interest per treatment at each week demonstrated clear separation of week 1 from week 4 and 7 expression profiles (Fig. 6). At week 1, the drench and foliar ANE treatments clustered together separately from the control, both showing elevated expression of most genes relative to the control. At weeks 4 and 7, the expression profiles were highly similar across all treatments, closely clustering together, apart from the drench treatment at week 4.

**Figure 7:**
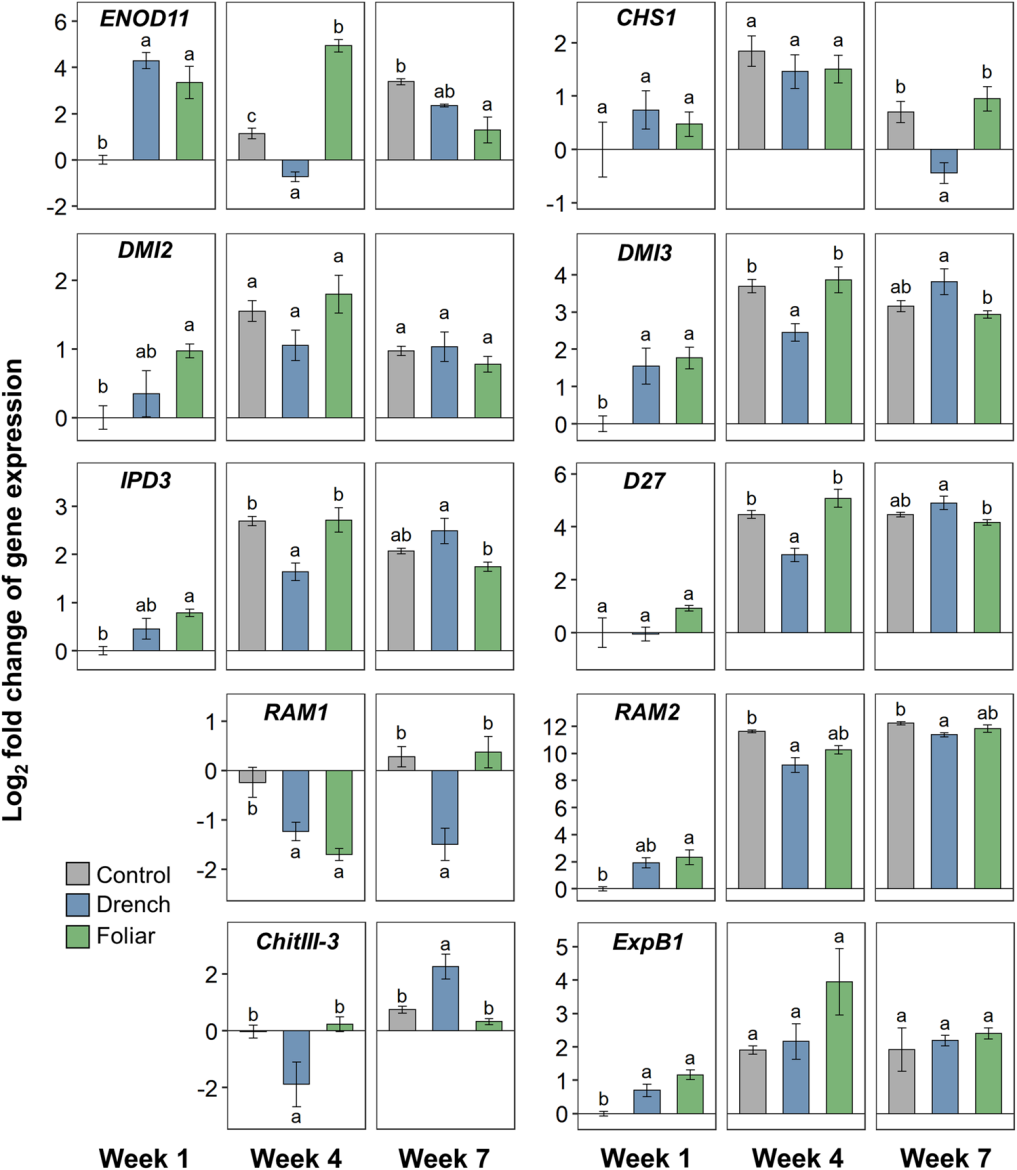
Expression of *ENOD11*, *CHS1*, *DMI2*, *DMI3*, *IPD3*, *D27*, *RAM1*, *RAM2*, *ChitIII-3*, and *ExpB1* in *M. truncatula* roots at week 1, 4 and 7 following inoculation with *R. irregularis*, as labelled. Values are the log_2_ fold change, ΔΔC_q_, compared to the control at week 1, or week 4 when expression was below detectable levels at week 1. Letters indicate Tukey’s post-hoc test significance groups, α = 0.05, following analysis of variance per week.

**Figure 8:**
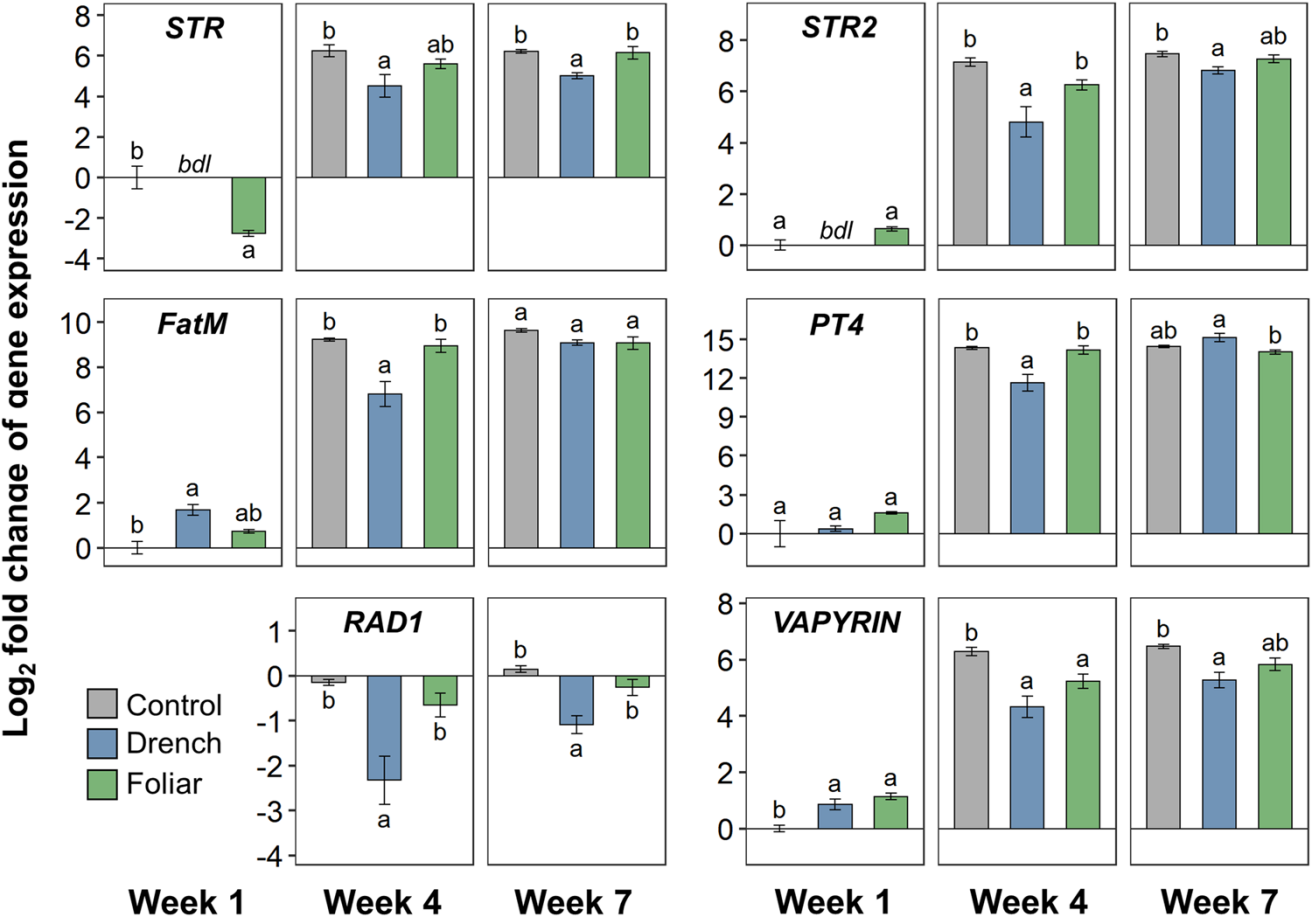
Expression of *STR*, *STR2*, *FatM*, *PT4*, *RAD1*, and *VAPYRIN* in *M. truncatula* roots at week 1, 4 and 7 following inoculation with *R. irregularis*, as labelled. Values are the log_2_ fold change, ΔΔC_q_, compared to the control at week 1, or week 4 when expression was below detectable levels at week 1. Letters indicate Tukey’s post-hoc test significance groups, α = 0.05, following analysis of variance per week; *bdl *below detectable level.

ANE treatment, drench or foliar, resulted in significant differences in expression of all genes examined at one or multiple weeks (Figs. 7 and 8). As was seen in the hierarchical clustering analysis (Fig. 6), the expression profiles of the ANE-treated plants were especially distinct from the control plants at week 1. At this time point, *ENOD11*, *DMI3*, *VAPYRIN*, and *ExpB1* showed significantly increased expression following both drench and foliar treatment compared to the control; *IPD3*, *RAM2*, and *DMI2* showed significant increases following foliar treatment; and *FatM* showed significantly increased expression following drench treatment (Figs. 7 and 8). The largest of differences in expression in week 1 was seen with *ENOD11*, showing 19.6-fold (4.29 log_2_-fold) increased expression in the drench-treated plants compared to the control. *STR* was the only gene with significantly lower expression compared to the control at week 1 (Fig. 8).

Expression profiles of the control and ANE-treated plants more closely resembled each other by week 4 and 7 (as demonstrated in the dendrogram, Fig. 6), with generally elevated expression levels of all mycorrhization-related genes in all three treatments (Figs. 7 and 8). Notable exceptions were *RAM1* and *RAD1*, which did not show increased expression over the course of weeks 4 and 7 (Figs. 7 and 8), and in fact demonstrated suppression in the ANE-treated plants, especially the ANE drench-treated plants; as well as *ChitIII-3*, for which the ANE drench-treated plants showed significantly lower expression at week 4 followed by increased expression at week 7, with an approximate fourfold increase in each case (Fig. 7). Expression of *ENOD11* fluctuated considerably, sustaining a high level of expression in the foliarly treated ANE plants at week 4, while dropping below the expression level of the control plants for the drench-treated ANE plants. By week 7, *ENOD11* expression was highest in the control plants (Fig. 7).

## Discussion

We demonstrate in the present work that an aqueous alkaline extract of the brown seaweed *A. nodosum* stimulated germination and development of the agronomically important arbuscular mycorrhizal fungus *R. irregularis*. The treatments also enhanced colonization and arbuscular maturation in *M. truncatula*. While earlier work demonstrated the stimulatory effect of methanolic seaweed extracts on AMF germination and growth^19,20^, crude methanolic extracts were notably shown to have no stimulatory effect, requiring further fractionation by flash chromatography to obtain bioactive fractions. More recent work showed that a neutral aqueous extract of the brown seaweed *Padina gymnospora* stimulated the colonization of *R. irregularis* in tomato plants^21^, but no direct effect of the seaweed extract on fungal spore germination and growth was reported. In contrast, we establish in the work reported here the direct stimulatory effect of whole aqueous alkaline *A. nodosum* extract on AMF spore germination, germ tube development, and hyphal branching. Such stimulation of hyphal growth and branching, a critical step in plant–AMF symbiosis establishment^28^, could increase the likelihood of contact and subsequent establishment of symbiosis between AMF and host plants, as well as improving soil quality by supporting the establishment of a cohesive mycelial network. Improving AMF establishments thus presents another potential agronomic benefit to the use of ANE as a biostimulant, influencing not only the plant, but the holobiont as a whole.

*A. nodosum* extracts are complex mixtures of numerous components, including mannitol, sterols, phlorotannins, flavonoids, minerals, and polysaccharides such as alginate and fucoidan as well as their extraction-induced derivatives, such as mono- and dicarboxylic acids^1,29^. The nature and quality of the raw material, as well as the conditions of the extraction process, are known to strongly influence the efficiency and selectivity of the extraction of these components, as well as their derivatization, and thus the ultimate bioactivity of the extract^30^. It is therefore perhaps not surprising that the aqueous alkaline extract used in the present work demonstrated stimulatory bioactivity of AMF development distinct from extracts produced using other extraction processes (such as methanolic extraction) or from other seaweed species.

Alginate-derived oligosaccharides^31^, phlorotannins^32^ and mannitol^33^ have previously been demonstrated to stimulate AMF growth. Fucoidan oligosaccharides and derivatives as well as sugar alcohols other than mannitol contained in *A. nodosum* may have similar effects^33^. In addition, various flavonoids have been identified in *A. nodosum*, including hispidulin, as well as gallocatechin and acacetin derivatives^29^, the application of which could further provide stimulatory effects. Flavonoids are known to have important roles in the signalling, establishment, and regulation of mycorrhizal endosymbiosis^34^, and their exogenous applications have been shown to induce hyphal growth, as well as enhancing plant root colonization^35–37^. Beyond flavonoids, Agregán et al.^29^ identified various other phenolic acids in extracts of *A. nodosum*, including hydroxybenzoic, quinic, and rosmarinic acid derivatives. Of these, low concentrations of *p*-hydroxybenzoic acid have previously been found to be stimulatory toward AMF spore germination^38^. Coumarins present another class of molecules capable of stimulating AMF colonization^13^. While having been identified in green algae^39^, to our knowledge to date they have not been discovered in brown algae in general nor *A. nodosum*. Likewise, strigolactones have been detected in select charophyte green algae, but not in other green algae nor brown algae^40^. Given the diverse molecular nature of ANE, the stimulatory effect of aqueous alkaline *A. nodosum* extract on AMF growth observed here could be due to a combined effect of multiple components in the extract, the nature of which requires further examination. Given the strong influence of the nature of the raw seaweed material and the extraction process on extract bioactivity, the degree to which the observed stimulation of AMF is specific to aqueous alkaline extracts of *A. nodosum* in particular warrants additional work. On the other hand, some of the stimulatory components in *A. nodosum* extracts such as alginic acid and fucoidan have been shown to become inhibitory of AMF growth at high concentrations^33^. This demonstrates the dose-dependent nature of ANE stimulation of AMF growth, requiring refinement of the application dose to ensure optimal modulation of the molecular communication between AMF and their plant hosts.

Stimulation of plant growth following application of ANE, as also observed here with *M. truncatula*, has been well documented in the literature^2–4^. Less well understood is how foliar application of seaweed extract could influence plant–microbe signaling and the structure of the rhizospheric microbiome. Foliar application of nutrients, hormones, or pathogens^24,41,42^ has been shown to result in systemic responses that modulate rhizospheric microbial community structure. In the present study, foliar ANE application did not result in statistically significant increases in mature mycorrhization, but did elicit expression of mycorrhizal accommodation-related genes in the distal *M. truncatula* roots. This suggests that the foliar application of ANE elicited a systemic response that activated the plant’s transcriptional symbiont accommodation response, but, presumably due to a lack of the direct stimulation of AMF germination and development afforded by soil drench applications, biostimulation with foliar applications did not ultimately result in statistically significant increases in colonization of plant roots.

Prior to contact with AMF in the soil, plants recruit and induce spore germination and growth of AMF through the exudation of molecular signals, namely flavonoids and strigolactones. The chalcone synthase CHS^43^ and the chloroplastic beta-carotene isomerase D27^44^ respectively are key enzymes in the biosynthetic pathways of these signaling molecules. In return, AMF exude diffusible lipochitooligosaccharide signals that, when detected by plants, initiate a signaling cascade in which the receptor kinase DMI2^45^ plays a central role to initiate a nuclear calcium spike, facilitated by the DMI1 cation channel^46^ and the downstream signaling kinase DMI3 ^47^, leading to activation of the transcriptional activator IPD3^48^. The calcium oscillations and ensuing transcriptional events are thought to trigger the cytoskeleton changes and host reprogramming events that enable accommodation of AMF. Various proteins facilitate this process, including: the expansin ExpB1^49^ and subtilase SBT1.2^50^ that help loosen cell walls to allow fungal entry; ENOD11 ^51^, a cell wall protein that facilitates prepenetration assembly formation and entry of fungal hyphae; and the transcription factors RAM1^52^ and RAD1^53^ that help orchestrate AMF accommodation.

Later phases of colonization are characterized by the presence of arbuscules, which are the sites of nutrient exchange between plant and AMF. Plants form specialized periarbuscular membranes around the AMF arbuscules to facilitate transfer of products, such as lipids produced by plants for AMF using enzymes such as the palmitoyl-acyl thioesterase FATM^54^ and the glycerol-3-phosphate acyl transferase RAM2^55^, and transferred across the periarbuscular membrane by the STR/STR2 proteins^56^. Phosphate scavenged by the AMF are taken up by the phosphate transporter PT4^57^, while VAPYRIN proteins^58^ aid nutrient exchange through facilitating endosomal trafficking. Throughout the lifecycle of an AMF-colonized plant cell, the biotic defense pathway needs to be suppressed; especially during late colonization, the chitinases CHITIII-2^59^ and CHITII-3^60^ are thought to be involved in this process.

ANE treatment particularly influenced gene expression at the early stages of colonization. The expression of a number of genes critical to the initial establishment of the plant–AMF symbiosis showed significantly elevated levels in both foliarly treated and drench-treated plants. These included genes required for symbiont accommodation program signaling—the signaling kinase-encoding genes *DMI2* and *DMI3*, and the transcriptional activator-encoding *IPD3*—as well as genes involved in arbuscule establishment: the glycerol-3-phosphate acyl transferase gene *RAM2*, the beta-expansin gene *ExpB1* and the periarbuscular membrane nutrient exchange-associated gene *VAPYRIN*. Expression of *ENOD11*, typically used as a marker of early colonization^51^, likewise demonstrated ANE-induced enhancement of symbiosis establishment at week 1. In later weeks however, expression of *ENOD11* was higher in the control plants, especially at week 7, in which its expression was higher than in both drench and foliar ANE-treated plants. Given that *ENOD11* is expressed transiently at sites of hyphal penetration^61^, it may be that *ENOD11* expression dropped at later stages of colonization after sufficient transient transcriptional stimulation had occurred in the ANE-treated plants. Meanwhile, expression of the transcription factor genes *RAM1* and *RAD1* showed suppression compared to the control plants, especially following drench ANE treatment. The reason why ANE drench-treated plants nonetheless displayed increased mycorrhization despite reductions in the expression of these important AMF accommodation-regulating transcription factors remains unclear, but may point to a rebalancing of the AMF accommodation transcriptional program in the presence of ANE. While the effect of drench ANE treatment on gene expression could conceivably be attributed to there being more rapid colonization following direct stimulation of AMF growth, the fact that foliar stimulation had a similar influence on gene expression in distal root tissue strongly suggests that ANE also stimulates a systemic plant response influencing AMF accommodation-related gene expression.

In comparison to the considerable differences in gene expression profiles between the ANE-treated and control plants at week 1, gene expression profiles generally resembled each other across the treatments at the later stages of colonization. This suggests that ANE treatment, whether foliar or via drench application, particularly exerted a differentiating influence on the earlier stages of mycorrhizal colonization. A notable exception, in addition to *RAM1* and *RAD1* as discussed earlier, was the gene *ChitIII-3*, a class III chitinase thought to be involved in the suppression of defence responses through degradation of fungal chitin elicitor molecules^59,60^. The ~ 4-fold increased expression of *ChitIII-3* in the drench ANE-treated plants can perhaps at least partially explain the high level of mature mycorrhization in these plants by week 7.

Prior to physical contact with AMF, plants exude flavonoids and strigolactones into the soil to signal for and stimulate growth and branching of AMF^26^. Chalcone synthase (encoded by CHS1) catalyzes the first step in biosynthesis of flavonoids in *M. truncatula*^43^, and its counterpart in strigolactone biosynthesis is the chloroplastic beta-carotene isomerase encoded by *D27*^44^. Expression of either gene was not significantly influenced by ANE treatment. It is possible that ANE drench treatment instead exerted its AMF colonization-promoting influence through molecular stimulation of AMF via the flavonoids and phenolic acids contained therein^29^, combining direct and indirect stimulation of the AMF–plant host system.

## Conclusions

The results presented here demonstrate that ANE facilitates enhanced establishment of plant–AMF symbiosis through both direct stimulation of AMF spore germination and hyphal growth, and through elicitation of the AMF accommodation program by the plant, enabling more rapid colonization and maturation of mycorrhization. This implies that, in addition to the well-documented direct elicitation of plant stress resilience, aqueous alkaline *A. nodosum* extracts can also elicit the indirect stress-protective functions of mycorrhizal symbiosis, representing another level of agronomic benefit accompanying the use of this class of biostimulants.

## Methods

### Fungal and plant material

*M. truncatula* cv. Jemalong A-17 seeds were obtained from the US National Plant Germplasm System (Accession PI 670016) and grown in accordance with and permission afforded under the US NPGS Distribution Policy. Two formulations of *Rhizophagus irregularis* isolate DAOM-197198 spores obtained from Premier Tech (Rivière-du-Loup, QC) were used: an aseptic liquid suspension (~ 400 spores per ml) for in vitro trials, as well as a dry kaolin-based powder formulation (~ 6400 spores per g) for greenhouse trials. The aseptic *R. irregularis* spore solution was kept refrigerated in the dark at 4 °C when not in use.

### In vitro* R. irregularis* sporulation assay

In vitro growth of *R. irregularis* spores was tested on 20-ml Petri plates with five different autoclaved media containing 15 g/l agar, replicated six times: ultrapure water; nutrient solution equivalent to the mineral nutrient content of 0.1 g/l *ANE* (Supplementary Table S1; based on the Long Ashton nutrient solution^62^); 1.0, 0.5, and 0.1 g/l *Ascophyllum nodosum* aqueous alkaline extract powder (batch #170-846; Acadian Seaplants, Darmouth, NS). *R. irregularis* spore suspension was added dropwise evenly to the sections of a 3 × 3 grid to a total of > 80 spores per plate and incubated at 25 °C in the dark.

At day 4 and 14, spore germination was counted using a microscope at 40 × magnification, expressed as the percentage of spores that had produced a germ tube^63^. Two photos of germinated spores were taken at random of each of the 9 sections on the plate and analyzed with ImageJ software (v.1.52n) to determine the length of the longest germ tube. Hyphal branching was additionally determined on day 4 for the nutrient control and 0.1 g/l ANE plates.

### Greenhouse trial

Seeds of *M. truncatula* cv. Jemalong A-17 were mechanically scarified using coarse grit sandpaper before seeding in a covered 200-cell plastic tray filled with moist potting medium (Pro-Mix BX, Premier Tech) watered as needed in a controlled environment room under a 16/8 h light/dark regime, at 25 °C and 60% relative humidity. Two weeks later seedlings were transplanted to 3 × 2 cell packs filled with golf course-grade mycorrhizae-free sand (Shaw Resources, Cambridge, NS) in the center of each cell in a shallow pit inoculated with 0.05 g *R. irregularis* powder formulation (~ 320 spores per cell). Nine cell packs per treatment (n = 54, N = 162) were placed on a greenhouse bench using randomized complete block design, rotated daily, using a 16/8 h light/dark regime at 60% relative humidity and 23.5 °C. All plants received 20 ml of a 1.0 g/l 14-0-14 NPK fertilizer weekly for the duration of the trial. Plants were treated the day after transplant and every 14 days afterwards as follows: (a) control plants received a 20-ml water drench; (b) ANE drench-treated plants received a 20-ml drench of 1 g/l ANE; (c) ANE foliarly treated plants received a 4-ml spray of 1.0 g/l ANE administered 30 cm from the plant with a spray bottle, and a 20 ml water drench. These application rates and volumes were found to be optimal for biostimulation of *M. truncatula* as assessed by plant phenotypic properties in our previous work (Hines, unpublished data), and are in line with recommended field application programs of ANE^64,65^. During foliar treatment, aluminum foil was wrapped around the stem of the plants to prevent treatment liquid from penetrating the substrate, isolating its application to the foliar tissue.

At weeks 3, 4, 6 and 8, ten random plants of the control, foliar and drench treatments were collected. Leaves were scanned and images analyzed for total leaf area on weeks 4 and 8 to examine early and late leaf growth (winFOLIA v.2019). Roots from the week 3, 4, 6 and 8 samples were cut into 2–4 cm segments and stored in a scintillation vial submerged in 50% alcohol and kept at 5 °C ahead of staining as previously described^66^. From each plant, fifty 1.5–2 cm root segments were examined under a compound microscope at 40 × magnification for the presence of mycorrhizal fungi, the colonization intensity, and the level of arbuscule abundance according to the method by Trouvelot et al.^25^. Accordingly, colonization intensity was scored from 1–5, with 0 indicating no colonization and a 5 indicating > 90% colonization, and colonization maturity was scored from 0–3, with a 0 indicating a lack of arbuscules and a 3 indicating arbuscules throughout the root segment.

### Mycorrhizal accommodation-related gene expression analysis

*M. truncatula* plants were grown and inoculated with *R. irregularis* in an identical manner and in parallel with the greenhouse trial described above. At 1, 4 and 7 weeks after inoculation, root samples were collected from 15 plants per treatment, pooled into 3 biological replicates of 5 plants, and flash frozen in liquid nitrogen. Root tissue was finely ground in a mortar and pestle containing liquid nitrogen and stored at − 70 °C until further use.

RNA was extracted from 150 mg ground root tissue and DNase-treated according to the method described by Oñate-Sánchez and Vicente-Carbajosa^67^. LiCl precipitation was additionally performed following extraction to eliminate polysaccharide contamination by addition of one-half volume 7.5 M LiCl and incubation at − 20 °C for 30 min, followed by 15 min centrifugation at 17k×*g* and washing of the pellet with 70% ethanol before air drying and dissolution in molecular biology-grade water. RNA concentration and purity was assessed through spectrophotometry (NanoDrop One, Thermo Scientific). RNA integrity was assessed using a Tris–acetate–EDTA denaturing gel (1% agarose, 0.64 g/l sodium hypochlorite, 100 V, 30 min) stained with SYBR Safe dye (Life Technologies).

The expression of nineteen genes of interest (Table 1) was analyzed using qPCR at week 1, 4 and 7. PCR primers were designed using Primer-BLAST (NCBI) and ordered from Integrated DNA Technologies (Supplementary Tables S2 and S3). PCR on the DNase-treated RNA samples using the *PTB* and *ENOD11* primer sets (no-reverse transcription controls) showed no amplification through 40 cycles. cDNA was synthesized from 0.8 µg RNA using oligo(dT)_20_ primers using the SuperScript III reverse transcriptase (Invitrogen). qPCR was conducted on a QuantStudio 3 system (Applied Biosystems) in triplicated 10-µl reactions containing 5 µL SYBR Green master mix (PowerUp, Applied Biosystems), 2 µl of 1.5 µM forward and reverse primer mix, 2 µl of 1 ng/µl cDNA template, and 1 µl H_2_O. The manufacturer’s recommended cycling conditions were followed, using an annealing temperature of 60 °C. Gene expression was analyzed using the comparative C_T_ method^68^, normalized to the geometric mean of two reference genes *PTB* and *PP2A*^69^, determined to be the most stable pair (*M* value of 0.639) among the reference genes tested here, which were selected from the work by Kakar et al.^70^ (Supplementary Table S2).

### Statistical analysis

Statistical significance was assessed within R (v. 3.6.0) using one-way analysis of variance with Tukey’s post-hoc test (α = 0.05) for spore germination extent, hyphal length, leaf area, and for ∆∆C_T_ values in the gene expression analysis. A *t* test (α = 0.05) was used for the hyphal branching data. The greenhouse plant root colonization data was assessed using a Kruskal–Wallis test followed by Dunn’s post-hoc test (α = 0.05) within R.

## Supplementary Information

Supplementary Tables.

## Data Availability

The datasets generated and analysed during the current study are available from the corresponding author on reasonable request.

## References

[1] Craigie JS (2011). Seaweed extract stimuli in plant science and agriculture. J. Appl. Phycol..

[2] Battacharyya D, Babgohari MZ, Rathor P, Prithiviraj B (2015). Seaweed extracts as biostimulants in horticulture. Sci. Hortic..

[3] Shukla PS (2019). *Ascophyllum nodosum*-based biostimulants: Sustainable applications in agriculture for the stimulation of plant growth, stress tolerance, and disease management. Front. Plant Sci..

[4] De Saeger J (2019). Toward the molecular understanding of the action mechanism of *Ascophyllum nodosum* extracts on plants. J. Appl. Phycol..

[5] Renaut S, Masse J, Norrie JP, Blal B, Hijri M (2019). A commercial seaweed extract structured microbial communities associated with tomato and pepper roots and significantly increased crop yield. Microb. Biotechnol..

[6] Wang M (2018). Responses of soil microbial communities to a short-term application of seaweed fertilizer revealed by deep amplicon sequencing. Appl. Soil Ecol..

[7] Bakker PAHM, Berendsen RL, Doornbos RF, Wintermans PCA, Pieterse CMJ (2013). The rhizosphere revisited: Root microbiomics. Front. Plant Sci..

[8] Badri DV, Vivanco JM (2009). Regulation and function of root exudates. Plant Cell Environ..

[9] Smith S, Read D (2008). Mycorrhizal Symbiosis.

[10] Sbrana C, Avio L, Giovannetti M (2014). Beneficial mycorrhizal symbionts affecting the production of health-promoting phytochemicals. Electrophoresis.

[11] Begum N (2019). Role of arbuscular mycorrhizal fungi in plant growth regulation: Implications in abiotic stress tolerance. Front. Plant Sci..

[12] Besserer A (2006). Strigolactones stimulate arbuscular mycorrhizal fungi by activating mitochondria. PLoS Biol..

[13] Cosme M, Fernández I, Declerck S, van der Heijden MGA, Pieterse CMJ (2021). A coumarin exudation pathway mitigates arbuscular mycorrhizal incompatibility in *Arabidopsis**thaliana*. Plant Mol. Biol..

[14] Pellegrino E (2012). Establishment, persistence and effectiveness of arbuscular mycorrhizal fungal inoculants in the field revealed using molecular genetic tracing and measurement of yield components. New Phytol..

[15] Cely MVT (2016). Inoculant of arbuscular mycorrhizal fungi (*Rhizophagus clarus*) increase yield of soybean and cotton under field conditions. Front. Microbiol..

[16] Rillig MC (2019). Why farmers should manage the arbuscular mycorrhizal symbiosis. New Phytol..

[17] Alam MZ, Braun G, Norrie J, Hodges DM (2014). *Ascophyllum extract* application can promote plant growth and root yield in carrot associated with increased root-zone soil microbial activity. Can. J. Plant Sci..

[18] Alam MZ, Braun G, Norrie J, Hodges DM (2013). Effect of *Ascophyllum* extract application on plant growth, fruit yield and soil microbial communities of strawberry. Can. J. Plant Sci..

[19] Kuwada K, Wamocho LS, Utamura M, Matsushita I, Ishii T (2006). Effect of red and green algal extracts on hyphal growth of arbuscular mycorrhizal fungi, and on mycorrhizal development and growth of papaya and passionfruit. Agron. J..

[20] Kuwada K, Ishii T, Matsushita I, Matsumoto I, Kazuomi K (1999). Effect of seaweed extracts on hyphal growth of vesicular-arbuscular mycorrhizal fungi and their infectivity on trifoliate orange roots. J. Jpn. Soc. Hortic. Sci..

[21] González-González MF (2020). Physiological, ecological, and biochemical implications in tomato plants of two plant biostimulants: Arbuscular mycorrhizal fungi and seaweed extract. Front. Plant Sci..

[22] El Boukhari M, Barakate M, Bouhia Y, Lyamlouli K (2020). Trends in seaweed extract based biostimulants: Manufacturing process and beneficial effect on soil-plant systems. Plants.

[23] Canarini A, Kaiser C, Merchant A, Richter A, Wanek W (2019). Root exudation of primary metabolites: Mechanisms and their roles in plant responses to environmental stimuli. Front. Plant Sci..

[24] Xiao H, Rodrigues RR, Bonierbale M, Veilleux R, Williams M (2018). Foliar application of Fe resonates to the belowground rhizosphere microbiome in Andean landrace potatoes. Appl. Soil Ecol..

[25] Trouvelot A, Kough J, Gianinazzi-Pearson V, Gianinazzi-Pearson V, Gianinazzi S (1986). Estimation of vesicular arbuscular mycorrhizal infection levels. Research for methods having a functional significance. Physiological and Genetical Aspects of Mycorrhizae.

[26] Choi J, Summers W, Paszkowski U (2018). Mechanisms underlying establishment of arbuscular mycorrhizal symbioses. Annu. Rev. Phytopathol..

[27] MacLean AM, Bravo A, Harrison MJ (2017). Plant signaling and metabolic pathways enabling arbuscular mycorrhizal symbiosis. Plant Cell.

[28] Akiyama K, Matsuzaki KI, Hayashi H (2005). Plant sesquiterpenes induce hyphal branching in arbuscular mycorrhizal fungi. Nature.

[29] Agregán R (2017). Phenolic compounds from three brown seaweed species using LC-DAD–ESI-MS/MS. Food Res. Int..

[30] Craigie JS, MacKinnon SL, Walter JA (2008). Liquid seaweed extracts identified using ^1^H NMR profiles. J. Appl. Phycol..

[31] Ishii, T. *et al.* Effects of alginate oligosaccharide and polyamines on hyphal growth of vesicular-arbuscular mycorrhizal fungi and their infectivity of citrus roots. in *9th Congress of the International Society of Citriculture* 1030–1032 (2000).

[32] Briand, X. & Salamagne, S. Use of phlorotannins as a stimulant for mycorrhizal and rhizobial symbioses. World patent WO2017/032954A1.

[33] Kuwada K (2005). Effect of mannitol from *Laminaria japonica*, other sugar alcohols, and marine alga polysaccharides on in vitro hyphal growth of *Gigaspora margarita* and root colonization of trifoliate orange. Plant Soil.

[34] Singla P, Garg N, Varma A, Prasad R, Tuteja N (2017). Plant flavonoids: Key players in signaling, establishment, and regulation of rhizobial and mycorrhizal endosymbioses. Mycorrhiza—Function, Diversity, State of the Art.

[35] Salloum MS (2018). Polyamines and flavonoids: Key compounds in mycorrhizal colonization of improved and unimproved soybean genotypes. Symbiosis.

[36] Siquiera JO, Safir GR, Nair MG (1991). Stimulation of vesicular-arbuscular mycorrhiza formation and growth of white clover by flavonoid compounds. New Phytol..

[37] Scervino JM (2007). The effect of flavones and flavonols on colonization of tomato plants by arbuscular mycorrhizal fungi of the genera *Gigaspora* and *Glomus*. Can. J. Microbiol..

[38] Fries LLM, Pacovsky RS, Safir GR, Siqueira JO (1997). Plant growth and arbuscular mycorrhizal fungal colonization affected by exogenously applied phenolic compounds. J. Chem. Ecol..

[39] Cotas J (2020). Seaweed phenolics: From extraction to applications. Mar. Drugs.

[40] Brewer PB, Koltai H, Beveridge CA (2013). Diverse roles of strigolactones in plant development. Mol. Plant.

[41] Carvalhais LC (2013). Activation of the jasmonic acid plant defence pathway alters the composition of rhizosphere bacterial communities. PLoS One.

[42] Berendsen RL (2018). Disease-induced assemblage of a plant-beneficial bacterial consortium. ISME J..

[43] Bonanomi A (2001). Arbuscular mycorrhiza in mini-mycorrhizotrons: First contact of *Medicago truncatula* roots with *Glomus intraradices* induces chalcone synthase. New Phytol..

[44] Liu W (2011). Strigolactone biosynthesis in *Medicago**truncatula* and rice requires the symbiotic GRAS-type transcription factors NSP1 and NSP2. Plant Cell.

[45] Calantzis C, Morandi D, Arnould C, Gianinazzi-Pearson V (2001). Cellular interactions between *G. mosseae* and a Myc-dmi2 mutant in *Medicago**truncatula*. Symbiosis.

[46] Peiter E (2007). The *Medicago truncatula* DMI1 protein modulates cytosolic calcium signaling. Plant Physiol..

[47] Catoira R (2000). Four genes of *Medicago truncatula* controlling components of a Nod factor transduction pathway. Plant Cell.

[48] Horváth B (2011). *Medicago truncatula IPD3* is a member of the common symbiotic signaling pathway required for rhizobial and mycorrhizal symbioses. Mol. Plant-Microbe Interact..

[49] Siciliano V (2007). Transcriptome analysis of arbuscular mycorrhizal roots during development of the prepenetration apparatus. Plant Physiol..

[50] Takeda N, Sato S, Asamizu E, Tabata S, Parniske M (2009). Apoplastic plant subtilases support arbuscular mycorrhiza development in *Lotus japonicus*. Plant J..

[51] Journet EP (2001). Medicago truncatula *ENOD11*: A novel RPRP-encoding early nodulin gene expressed during mycorrhization in arbuscule-containing cells. Mol. Plant-Microbe Interact..

[52] Gobbato E (2012). A GRAS-type transcription factor with a specific function in mycorrhizal signaling. Curr. Biol..

[53] Rey T (2017). The *Medicago truncatula* GRAS protein RAD1 supports arbuscular mycorrhiza symbiosis and *Phytophthora palmivora* susceptibility. J. Exp. Bot..

[54] Bravo A, Brands M, Wewer V, Dörmann P, Harrison MJ (2017). Arbuscular mycorrhiza-specific enzymes FatM and RAM2 fine-tune lipid biosynthesis to promote development of arbuscular mycorrhiza. New Phytol..

[55] Wang E (2012). A common signaling process that promotes mycorrhizal and oomycete colonization of plants. Curr. Biol..

[56] Zhang Q, Blaylock LA, Harrison MJ (2010). Two *Medicago truncatula* half-ABC transporters are essential for arbuscule development in arbuscular mycorrhizal symbiosis. Plant Cell.

[57] Harrison MJ, Dewbre GR, Liu J (2002). A phosphate transporter from *Medicago**truncatula* involved in the acquisition of phosphate released by arbuscular mycorrhizal fungi. Plant Cell.

[58] Bapaume L (2019). VAPYRIN marks an endosomal trafficking compartment involved in arbuscular mycorrhizal symbiosis. Front. Plant Sci..

[59] Salzer P (2000). Differential expression of eight chitinase genes in *Medicago truncatula* roots during mycorrhiza formation, nodulation, and pathogen infection. Mol. Plant-Microbe Interact..

[60] Bonanomi A, Wiemken A, Boller T, Salzer P (2001). Local induction of a mycorrhiza-specific class III chitinase gene in cortical root cells of *Medicago truncatula* containing developing or mature arbuscules. Plant Biol..

[61] Chabaud M, Venard C, Defaux-Petras A, Bécard G, Barker DG (2002). Targeted inoculation of *Medicago truncatula* in vitro root cultures reveals MtENOD11 expression during early stages of infection by arbuscular mycorrhizal fungi. New Phytol..

[62] Hawkins HJ, George E (1997). Hydroponic culture of the mycorrhizal fungus *Glomus**mosseae* with *Linum**usitatissimum* L., *Sorghum**bicolor* L. and *Triticum**aestivum* L. Plant Soil.

[63] Coelho, L. C. S., Mignoni, D. S. B., Silva, F. S. B. & Braga, M. R. Seed exudates of *Sesbania virgata* (Cav.) Pers. stimulate the asymbiotic phase of the arbuscular mycorrhizal fungus *Gigaspora albida* Becker & Hall. *Hoehnea* **46** (2019).

[64] Xu C, Leskovar DI (2015). Effects of *A. nodosum* seaweed extracts on spinach growth, physiology and nutrition value under drought stress. Sci. Hortic..

[65] Li Y, Mattson NS (2015). Effects of seaweed extract application rate and method on post-production life of petunia and tomato transplants. HortTechnology.

[66] Vierheilig H, Coughlan AP, Wyss U, Piché Y (1998). Ink and vinegar, a simple staining technique for arbuscular-mycorrhizal fungi. Appl. Environ. Microbiol..

[67] Oñate-Sánchez L, Vicente-Carbajosa J (2008). DNA-free RNA isolation protocols for *Arabidopsis thaliana*, including seeds and siliques. BMC Res. Notes.

[68] Schmittgen TD, Livak KJ (2008). Analyzing real-time PCR data by the comparative CT method. Nat. Protoc..

[69] Vandesompele J (2002). Accurate normalization of real-time quantitative RT-PCR data by geometric averaging of multiple internal control genes. Genome Biol..

[70] Kakar K (2008). A community resource for high-throughput quantitative RT-PCR analysis of transcription factor gene expression in *Medicago truncatula*. Plant Methods.

