# Supplementary material for: Alkaline extract of the seaweed Ascophyllum nodosum stimulates arbuscular mycorrhizal fungi and their endomycorrhization of plant roots
Source: Sci Rep. 2021 Jun 29;11:13491. doi: 10.1038/s41598-021-93035-9 (PMC8241850; doi:10.1038/s41598-021-93035-9)
Supplement: Supplementary file 1 — Supplementary Tables. [file 41598_2021_93035_MOESM1_ESM.docx]

**Alkaline extract of the seaweed Ascophyllum nodosum stimulates arbuscular mycorrhizal fungi and their endomycorrhization of plant roots**

Sarah Hines^1^, Timo van der Zwan^2^, Kevin Shiell^2^, Katy Shotton^2^, Balakrishnan Prithiviraj^1*^

^1^Marine Bioproducts Research Laboratory, Department of Plant, Food and Environmental Sciences, Dalhousie University, Truro, Nova Scotia, Canada

^2^Acadian Plant Health, Acadian Seaplants Ltd., Dartmouth, Nova Scotia, Canada

*Author for correspondence: bprithiviraj@dal.ca

**Table S1** Composition of nutrient solution used as nutrient equivalent of *A. nodosum* extract

| **Component** | **Concentration** (µM) |
| --- | --- |
| KNO_3_ | 400 |
| Ca(NO_3_)_2_·4H_2_O | 400 |
| MgSO_4_·7H_2_O | 150 |
| NaH_2_PO_4_·H_2_O | 266 |
| Ferric-citrate·H_2_O | 19 |
| MnSO_4_·H_2_O | 4 |
| CuSO_4_·5H_2_O | 1 |
| ZnSO_4_·7H_2_O | 4 |
| H_3_BO_3_ | 60 |
| Na_2_MoO_4_·2H_2_O | 1 |
| NaCl | 200 |
| CoSO_4_·7H_2_O | 0.38 |
| KCl | 1700 |
| KOH | 1700 |

**Table S2** List of primers for the reference genes used in this study and their characteristics

| **Gene symbol** | **Protein** | **Locus tag** | **Forward primer**  (5’ → 3’) | **Reverse primer**  (5’ → 3’) | **Amplicon length**(nt) | | **Efficiency** (%) | | **R^2^** |
| --- | --- | --- | --- | --- | --- | --- | --- | --- | --- |
| *PP2A* | protein phosphatase PP2A regulatory subunit A | MTR_6g084690 | GGTTAAGAGGCTGGCTTCTGG | GGCATATCATCTTGGCACAGC | 144 | 98 | | 0.99 | |
| *PPRep* | pentatricopeptide repeat-containing protein, mitochondrial | MTR_6g079920 | AAACTGAAACTATGGACAAACTGC | CATCATCACCACACTCACAGG | 82 | 104 | | 0.99 | |
| *PI4KG3* | phosphatidylinositol 4-kinase gamma 3 | MTR_3g091400 | TGGTTATTGCCTGCCCAAGA | TGGTGTCTAGGGAGTAAGGCT | 89 | 103 | | 0.96 | |
| *PTB* | polypyrimidine tract-binding protein homolog 2 | MTR_3g090960 | GAAGCATTGGAAGGACACTGC | GGCTGAGTGTTCACAACTGG | 149 | 100 | | 0.99 | |

**Table S3** List of primers for the genes of interest used in this study and their characteristics

| **Gene symbol** | **Protein** | **Locus tag** | **Forward primer**  (5’ → 3’) | **Reverse primer**  (5’ → 3’) | **Amplicon length**(nt) | | **Efficiency** (%) | | **R^2^** |
| --- | --- | --- | --- | --- | --- | --- | --- | --- | --- |
| *ENOD11* | cell wall repetitive proline-rich protein | MTR_3g415670 | TATGGTAACCAGCCTCCACC | AAGCATTGGTAAACCTTGTTGC | 71 | 101 | | 0.99 | |
| *CHS1* | chalcone synthase | MTR_7g016780 | GCAAATTGTGTTGAACAAAGCAC | TTGATCATGGATTTATCACACATGC | 116 | 94 | | 0.99 | |
| *DMI2* | receptor kinase | LOC11407326 | GCTTGGTTGAATGGGCTAAACC | ACTCTCCACAATGCCTCTGC | 103 | 112 | | 0.99 | |
| *DMI1* | nuclear ion channel for signal transduction | LOC11433851 | GCAGATCAGAGTGATGCACG | GGGTTCATTGTCTAGGTCGC | 111 | nd | | nd | |
| *D27* | beta-carotene isomerase, chloroplastic | MTR_1g471050 | ATCCAGCACTCAAGCAACCC | CTGACCTGAAAGCCTGCTGC | 95 | 93 | | 0.99 | |
| *IPD3* | transcription factor, “CYCLOPS” | MTR_5g026850 | CATGGAAACAAGTGGGAGAAAGC | CCATCTTGCCATCTGAAACAGC | 144 | 100 | | 0.96 | |
| *DMI3* | calcium and calcium/calmodulin-dependent serine/threonine-protein kinase | LOC11405240 | TGATGGAACAGTTGACATGCG | TCATACATCTGGAAGCACAAACG | 99 | 97 | | 0.95 | |
| *RAM1* | DELLA transcription factor | MTR_7g027190 | GCTAGTGCTTCTCTTTCACAAGC | GTCCACTGTCTTGCTCCTGC | 130 | 102 | | 0.97 | |
| *RAM2* | glycerol-3-phosphate 2-O-acyltransferase 6 | MTR_1g040500 | CATGGCTTTGTGCAAGGAGG | TTTGTCACTTGTCACTGTTGTTACC | 70 | 97 | | 0.98 | |
| *ExpB1* | expansin-like B1 | MTR_4g099400 | CGTGATCCTCTACGTTGGTGG | CCATTTGTGCTGTTCTTTCTGCC | 73 | 103 | | 0.98 | |
| *ChitIII-3* | acidic endochitinase | MTR_0027s0260 | CAGGCAGTGGCTTCATTCCT | ACCACAGCATAACACCGCC | 93 | 100 | | 0.99 | |
| *ChitIII-2* | acidic endochitinase | MTR_2g102020 | TGGAGAATTAGGCGTCGCAC | CCATCGCTTCCACGAATCCA | 127 | 100 | | 0.99 | |
| *STR* | ABC transporter G family member 20 | LOC11412901 | GACATAGCTCAGGGTGGTAGC | GAGTGTGAAGTGCATCTGGC | 139 | 98 | | 0.99 | |
| *STR2* | ABC transporter G family member 17 | LOC11407838 | AGCTCACAAGAACATCTAGGTCC | ATGTTTGTGAAGTTGCGGCG | 86 | 103 | | 0.99 | |
| *FatM* | palmitoyl-acyl carrier protein thioesterase, chloroplastic | MTR_1g109110 | CGACGAGACTGGCTCATAAGG | CATCACCCATGTGCTTGTTGC | 72 | 105 | | 0.97 | |
| *RAD1* | DELLA transcription factor | MTR_4g104020 | ACATGGACACCAATGGCGAG | CCTAAGCCTCCGAACACGG | 73 | 104 | | 0.99 | |
| *PT4* | inorganic phosphate transporter 1-11 | MTR_1g028600 | ACATGCCATGCTTTCAGTGC | TCTTCCTTGGCGTACCATCC | 93 | 101 | | 0.99 | |
| *VAPYRIN* | ankyrin repeat protein | MTR_6g027840 | ACAATTTATGTGCGGCTGCG | TGATCCCTTCCATTGATCACCC | 88 | 94 | | 0.99 | |
| *SBT1.2* | subtilisin-like protease | MTR_5g011310 | GTTTGTACTGATAACATTCCATGCG | GCAATTGGGTCTTCAAAGAATGG | 137 | 102 | | 0.99 | |

nd: not determined, expression levels were below limit of detection
